# Is there any relationship between nurses’ perceived organizational justice, moral sensitivity, moral courage, moral distress and burnout?

**DOI:** 10.1186/s12912-023-01536-x

**Published:** 2023-10-06

**Authors:** Sara Lotfi-Bejestani, Foroozan Atashzadeh-Shoorideh, Raziyeh Ghafouri, Malihe Nasiri, Kayoko Ohnishi, Fataneh Ghadirian

**Affiliations:** 1 Master of Science in psychiatric nursing student, School of Nursing & Midwifery, Shahid Beheshti University of Medical Sciences, Tehran, Iran; 2 Department of Psychiatric Nursing & Management, School of Nursing and midwifery, Shahid Beheshti University of Medical Sciences, Tehran, Iran; 3 Medical-Surgical Nursing Department, School of Nursing & Midwifery, Shahid Beheshti University of Medical Sciences, Tehran, Iran; 4 Department of Basic Sciences, School of Nursing & Midwifery, Shahid Beheshti University of Medical Sciences, Tehran, Iran; 5 Graduate School of Nursing, Nagoya City University, Nagoya, Japan; 6 Psychiatric Nursing and Management, School of Nursing and Midwifery, Shahid Beheshti University of Medical Sciences, Tehran, Iran

**Keywords:** Burnout, Mental Health, Moral courage, Moral distress, Moral sensitivity, Nurse, Perceived organizational justice

## Abstract

**Aim:**

The present study is an attempt to investigate the relationship between Corley’s model variables in mental health nurses.

**Background:**

Based on Corley’s model, burnout and moral distress in nurses are, in retrospect, the consequences of the interplay of organizational and individual factors such as perceived organizational justice, moral sensitivity, and moral courage. The relationship between two variables or three variables of Corley’s moral distress model has been investigated, but the test of Corley’s moral distress model with more variables has not been done. Therefore, this research was proposed with the aim of investigating the relationship between the variables of moral courage and moral sensitivity (as characteristics of nurses), perceived organizational justice (as an antecedent), moral distress, and job burnout (as consequences of moral distress).

**Methods:**

The study was conducted as a descriptive correlational study involving 500 nurses working in the mental health wards of hospitals. Data collection was conducted using perceived organizational justice scale, moral sensitivity scale, moral courage scale, moral distress scale, and burnout inventory.

**Results:**

The results showed a significant relationship between perceived organizational justice, moral sensitivity, moral courage, and moral distress (< 0.05). Moreover, perceived organizational justice and moral distress had an inverse relationship. Moral sensitivity and moral courage had a direct relationship with moral distress (< 0.05). Furthermore, the results showed inadequate model fitness.

**Conclusions:**

This study adds to the existing knowledge about the experiences of mental health nurses and their interactions with both organizational and individual factors. It highlights that the connections between perceived organizational justice, moral sensitivity, moral courage, moral distress, and burnout are intricate and multifaceted. As we deepen our understanding of these relationships, it opens the door for the development of interventions and strategies to enhance nurses’ well-being and the quality of care they deliver in mental health settings. Moreover, future research and ongoing refinement and expansion of Corley’s model will be crucial in addressing the complex challenges within the healthcare sector.

**Supplementary Information:**

The online version contains supplementary material available at 10.1186/s12912-023-01536-x.

## Background

Nurses in clinical settings often find themselves facing various moral conflicts in their professional roles, such as decision-making challenges, lack of autonomy, conflicts with physicians and institutional policies, deficient work infrastructure, weakened professional relationships, insufficient professional skills, and a lack of humanization [1–4]. These conflicts can lead to nurses experiencing moral distress (MD), which is a psychological and emotional response to situations that go against their moral principles [5]. In 2020, Berhie and colleagues asserted that MD manifests when individuals acknowledge the morally correct course of action, yet organizational limitations and apprehensions regarding potential consequences impede their ability to act accordingly [6]. In the U.S., one in three nurses experiences MD, and approximately 17.5% of newly hired nurses leave their jobs within the first year due to MD [7]. While the prevalence of MD among Iranian psychiatric nurses is unknown, the reported burnout rate among hospital nurses in Iran is 25%, with the highest prevalence (75%) observed among hospital nurses in Tehran [8].

**Fig. 1:**
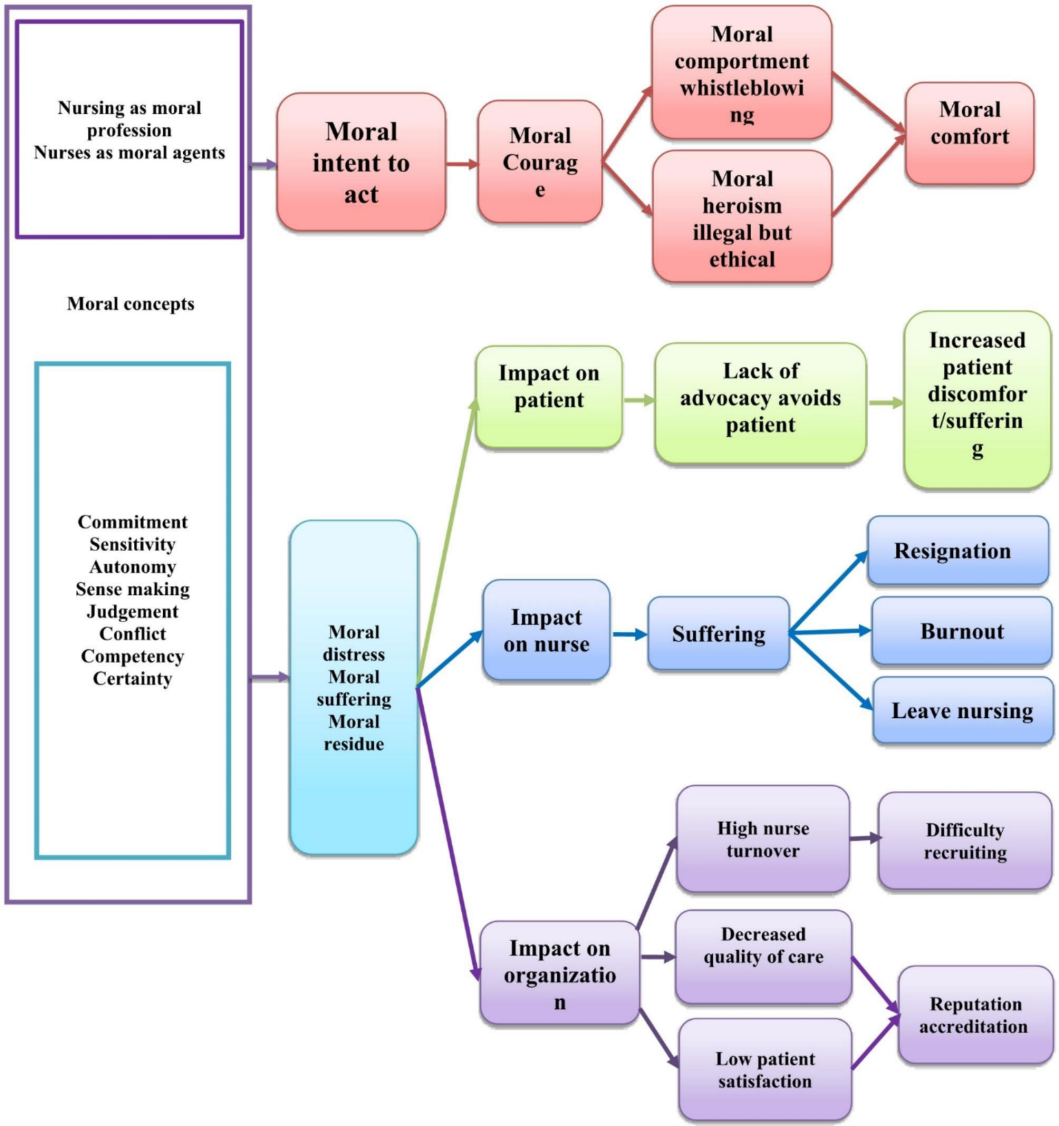
Corley’s model for a theory of moral distress

Research indicates that if left unaddressed, MD can result in a loss of motivation, desensitization, burnout, and a decline in patient safety and care quality [5]. The impact of MD extends beyond individual nurses, affecting the entire organization and the quality of patient care. Nurses, as moral agents, require specific qualities and prerequisites to navigate MD, make ethical decisions, and deliver high-quality care [9]. Corley proposed a model of MD with antecedents and consequences, as illustrated in Fig. 1. These concepts and their relationships were further refined by Morley et al. in 2019 [10]. This model explores ethical concepts and their relationships, emphasizing the connections between MD, moral courage (MC), moral sensitivity (MS), perceived organizational justice (POJ), and burnout [1].

Corley (2002) regarded burnout and MD in nurses as outcomes, suggesting that the interplay of organizational and individual factors, such as POJ, MS, and MC, serves as precursors for burnout and MD in nurses [1]. MC refers to the inner strength that empowers nurses as moral agents to act in alignment with ethical principles and convictions during ethical conflicts, even when facing potential negative consequences [11]. MS involves an awareness of how one’s actions may affect others, particularly in understanding the moral implications of one’s decisions in various situations within nursing [12]. POJ is a personal perception and evaluation of fairness or unfairness in the workplace [13]. An example of organizational justice is the fair distribution of rewards and incentives among employees. When employees perceive higher levels of justice, they exhibit greater commitment to their organization [14]. Organizational justice influences employees’ behavior, leading to outcomes such as satisfaction, trust, and organizational commitment [15, 16]. While other organizational climate factors contribute to shaping the ethical climate, we focus on POJ to provide a comprehensive analysis of justice-related perceptions affecting moral factors within organizations [17–19].

Working in mental health wards can exacerbate MD and burnout among nurses due to challenges such as patient disorientation, difficulties in decision-making, opposition from patients’ families, and resistance to treatment acceptance. A systematic review study identified burnout rates among mental health nurses, revealing that 25% experience high levels of emotional exhaustion, 15% exhibit depersonalization, and 22% report low personal accomplishment [20].

However, studies examining the relationship between MD, burnout, and the unique challenges faced by mental health nurses have yielded contradictory results, likely attributable to the complex nature of these phenomena and contextual variations within mental health settings. For example, a study by Ohnishi et al. (2010) identified “low staffing” as the most significant factor contributing to MD among Japanese psychiatric nurses and found that organizational factors significantly predicted burnout in this group [2]. López-López et al.‘s meta-analysis demonstrated low-to-moderate prevalence of burnout dimensions among mental health nurses, with MD influencing emotional exhaustion and depersonalization, while organizational factors played a role in personal accomplishment [20]. Additionally, Jansen et al. (2022) highlighted how cultural and political policies within psychiatric units could intensify MD among nurses, as they grapple with the challenge of reducing coercion while ensuring patient and staff safety and fostering a therapeutic environment [21].

Few studies have explored the factors of MD and burnout among mental health nurses, with many omitting burnout as an outcome measure. For instance, Haghighinezhad et al. (2019) found a significant negative correlation between POJ and MD [9]. Robaee et al. (2018) investigated the relationship between perceived organizational support and MD and concluded that while nurses reported low perceived organizational support and high MD, there was no direct relationship between these two variables, suggesting the presence of other influential factors [22]. Shoorideh et al. (2015) identified a positive relationship between MD and burnout, as well as between burnout and the intention to quit, though no statistical relationship was found between MD and nurses’ intention to leave their service [3]. Additionally, Fumis et al. (2017) established a relationship between MD and severe burnout [23]. While some studies have examined pairs of variables based on Corley’s model [9, 22, 24], no comprehensive investigation involving multiple variables simultaneously has been conducted. This study aims to explore the relationships among POJ, MS, MC, MD, and burnout among nurses working in mental health wards (see Table 1).

**Table 1:** Variables of research model

characteristic of nurses	antecedents	consequences
Moral courage Moral sensitivity	Perceived organizational justice	Moral distress Job burnout

**Fig. 2:**
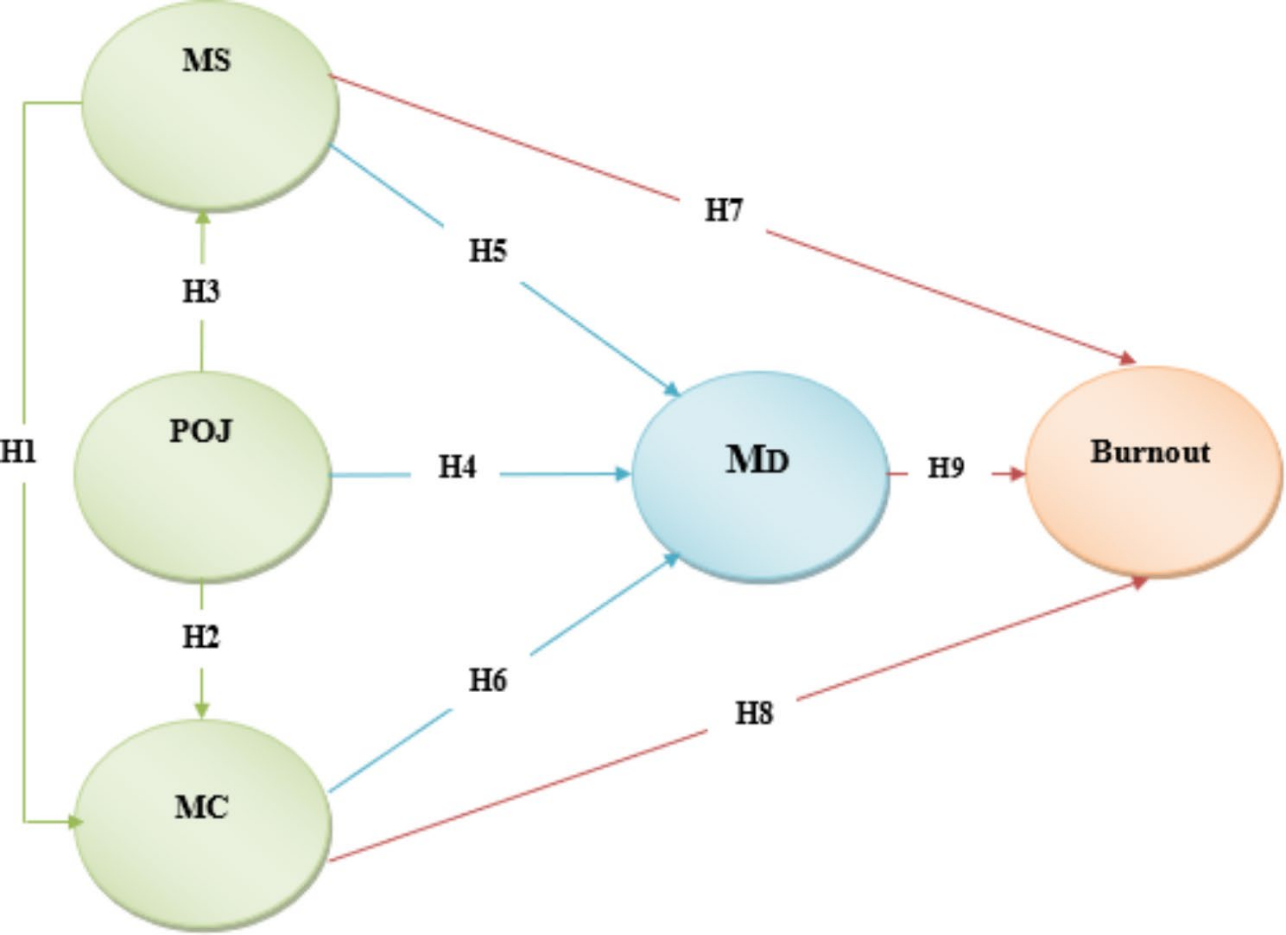
A hypothesized path model of the proposed research model. POJ; Perceived Organizational Justice, MS; Moral sensitivity, MC; Moral Courage, MD; Moral Distress

Understanding burnout and MD among mental health nurses within the intricate web of relationships between individual factors (MC and MS) and organizational factors (POJ) can enhance our understanding of complex ethical dynamics in psychiatric mental health nursing. In this study, we employ path analysis to uncover the interconnections between POJ, MS, MC, MD, and burnout among nurses working in mental health wards. Figure 2 illustrates the hypothetical path model of MD and burnout, incorporating MC, MS, and POJ.

## Methods

### Objectives

This descriptive correlational study aimed to offer an overview of the present status of POJ, MC, MS, as well as burnout and MD within the context of nurses employed in mental health wards. Additionally, it sought to investigate the associations among these variables, considering POJ as an antecedent, MC and MS as individual characteristics of nurses, and MD and job burnout as outcomes attributed to MD. To achieve these objectives, path analysis was employed.

### Study design and setting

The study was conducted as a descriptive correlational study. The data were collected between May 2020 and September 2020 in the mental health wards of hospitals in different states of Iran. The research instrument was provided to 700 members of the study population and 510 of them completed the questionnaire (responsiveness = 70%). Questionnaires which were not fully completed were excluded (n = 10).

### Participants

Nurses were recruited from hospitals having psychiatric wards in different regions of Iran. There were 18 hospitals with a psychiatric department around the 31 states. Participants were approached personally by involving the nursing supervisors. All nurses who worked in the study settings were asked to participate for screening. Because experience working with psychiatric patients and academic training might have an impact on attitudes toward right action and moral constraints, inclusion criteria were established. Inclusion criteria were having at least one year of work experience, and having B.Sc. degree or above in nursing. Based on diagnostic criteria, stress-induced maladaptive responses usually resolve within 6 months, although this time period may be longer for some personality traits [25]. No exclusion criteria (other than the opposite of the inclusion criteria) are specified. Subjects recruited using the following methods: First, an email distributed to all nurses. This email briefly informed the nurses about the study and invited them to click on the link to participate. Nurse administrators in each of these sites also personally informed staff about the study.

To prevent participation from those who did not meet the inclusion criteria, a question to screen viewers appeared initially when a person clicks on the link (i.e., “I am RN and have been working as a nurse in direct patient care for at least one year.”) Response options include *Yes* and *No*. If a response is No, then they will receive a screen that thanks them for their interest and informs them that they will not be allowed to continue.

Persons who met the inclusion criteria were then directed to a screen that provides the letter informing them about the study, its risks and benefits, and how their responding to items will infer consent. They were informed that if they submit responses, this would indicate voluntary participation.

### Sample size calculation

Sample size was calculated for structural equation modeling. The sample size was computed using the unconditional power calculation method. A sample size of 489 achieves 80% power to detect a partial ρ² of at least 0.30 attributed to two independent variable(s) when the significance level (alpha) is 0.05 and the actual value of ρ² is 0.40 after adjusting for and adjusted for 30% dropout rate.

### Data collection

The required data were collected using a demographic form, the POJ questionnaire, the MS scale, the MC scale, the MD scale, and the burnout inventory. The Persian version of all questionnaires has been prepared and psychometric evaluations have validated the instruments and have been used in many Iranian studies [26–30]. The demographic form included age, gender, marital status, education level and working years in the mental health ward.

### Instruments

#### POJ questionnaire

The questionnaire was designed by Niehoff and Moorman (1993) with 20 items and three dimensions, namely distributive justice (five items), procedural justice (six items) and interactive justice (nine items). The POJ score is the sum of the scores of these three subscales. The items are structured based on a five-point Likert scale (1 = strongly disagree, …, 5 = strongly agree). The range of scores for distributive justice is 5–25, for procedural justice 6–30, and for interactive justice 9–45. The range of scores in the questionnaire is between 20 and 100, and dividing the score by 20 gives a number between 1 and 5. The higher an individual’s score, the more pronounced the POJ [9, 31]. In the current study, Cronbach’s alpha for the POJ was determined to be 0.85, while the ICC method demonstrated a stability of r = 0.87 for this tool.

#### MS scale

Han et al. (2010) designed the MS scale with 25 items that include three dimensions, namely respect for the patient, professional responsibility, and ethical behavior. The items are five-alternative questions (strongly agree, …, strongly disagree) with scores ranging from 0 to 4. The range of scores for respect for the patient is 0–32, for professional responsibility 0–32, and for ethical behavior 0–36. The total score of the instrument ranges from 0 to 100 [32]. This scale was translated and validated by Mohammadi et al. in 2017 in Iran. The mean scores of each aspect and total score were considered as the score of MS. The total scores of 0–50, 50–75, and 75–100 for each participant were regarded as low, intermediate, and high MS respectively [33]. In the current study, the Cronbach’s alpha for the overall MS scale was determined to be 0.90, while the ICC method indicated a stability of r = 0.86 for this tool.

#### MC scale

Sekerka et al. designed the scale MC with 15 items, which include five dimensions namely moral agency, multiple values, endurance of threats, going beyond compliance, and moral goals. The items are designed based on Likert seven-point scale (never right, …, always right), which are scored from 1 to 7. The score range of each subscale ranges from 3 to 21 and the total score of the instrument ranges from 15 to 105. A score ranging from 15 to 50 indicates a low level of MC. A score in the range of 51 to 75 signifies an average level of MC, while a score between 76 and 105 indicates a high level of MC [34]. In the present study, the tool exhibited a Cronbach’s alpha value of 0.77, while the internal consistency, assessed using the ICC method, was found to be r = 0.85.

#### MD scale

The scale contains 15 items and three factors, namely acquiescence to patients’ rights violations (six items), low staffing (five items) and unethical conduct by caregivers (four items). The instrument was developed by Ohnishi et al. in 2018. The items are on a seven-point Likert scale (0 = not distressed or not experienced, …, 6 = distressed intensely). The range of scores of the instrument is from 0 to 90. A summary score is calculated by adding up the scores of the 4 to 6 items within each factor, where a higher score indicates a greater degree of MD [2, 27, 35]. In present research, the average inter-item correlations (AIC) for acquiescence to patients’ rights violations, unethical conduct by caregivers, and low staffing factors were 0.65, 0.62, and 0.62, respectively. The Cronbach’s alpha values for these factors and the total scale were 0.90, 0.82, 0.82, and 0.82, respectively. These results indicate that both the AIC and Cronbach’s alpha fall within the acceptable range, affirming the instrument’s reliability among the agents.

#### Burnout inventory

Copenhagen Burnout Inventory was developed by Kristensen in 2005 with 19 items [36]. The validity of the instrument was confirmed with four dimensions of personal burnout (seven items), nature work-related burnout (three items), work aversion-related burnout (three items) and client-related burnout (six items).

The scoring scale for personal burnout spans from 0 to 700. Values in the range of 0 to 233 indicate a low level of burnout, while values between 234 and 466 signify a moderate level, and values falling between 467 and 700 correspond to a high level. For nature work-related burnout, the scoring scale ranges from 0 to 300. Values from 0 to 100 represent a low level of burnout, those between 101 and 200 signify a moderate level, and values falling between 201 and 300 correspond to a high level. The scoring scale for work aversion-related burnout also extends from 0 to 300, with values from 0 to 100 representing a low level of burnout, values between 101 and 200 signifying a moderate level, and values within the range of 201 to 300 corresponding to a high level. Client-related burnout is scored on a scale of 0 to 600. Values from 0 to 200 represent a low level of burnout, while values between 201 and 400 indicate a moderate level, and values ranging from 401 to 600 correspond to a high level. Each aspect’s score is presented as the mean value within that aspect, and no total score of burnout is provided. Higher scores on each sub-scale indicate a greater degree of burnout [36–38]. In the present study, the internal consistency, as indicated by the alpha coefficient, was high at 0.95. Additionally, the intra class correlation (ICC) scores for various dimensions of burnout were as follows: 0.95 for personal burnout, 0.84 for nature work-related burnout, 0.83 for work aversion-related burnout, and 0.90 for client-related burnout. Furthermore, the Cronbach’s alpha coefficient for the tool used in this study was 0.78.

### Data analysis

Data analyses were performed in SPSS 21 using Pearson’s correlation coefficient. To examine the relationship between POJ, MS, MC, MD, and burnout simultaneously, a structural equation modelling (SEM) approach using the maximum-likelihood estimation was applied in this study by LISEREL (8.80). The confidence interval and statistically significant were set at 95% and p < 0.05, respectively. The goodness of fit of the SEM model was assessed using the chi-square statistic, the comparative fit index (CFI), the goodness-of-fit index (GFI) and the root mean square of approximation (RMSEA). The cut off values for the SEM model used in the current analysis were: a p-value of the chi-square statistic > 0.05, a value of > 0.95 for CFI, GFI and < 0.08 for RSMEA.

## Results

### Demographic characteristics of the participants

This study involved 500 participants, and the mean age of the participants was 37.59 years (SD = 7.03) and the rest of demographic information is shown in Table 2. Most participants were female (n = 377, 75.4%), single (n = 289, 57.8%), had a bachelor’s degree (n = 426, 85.25%), and had worked in mental health wards for 5–15 years (n = 250, 50%).

**Table 2:** Demographic characteristics of the participants (n = 500)

Demographics	Range	n(%)
**Age**	23–32 years	96 (19.2)
	33–42 years	260 (52)
	43–52 years	110 (22)
	52–63 years	13 (2.6)
	Missing data	21 (4.2)
**Gender**	Male	112 (22.4)
	Female	377 (75.4)
	Missing data	11 (2.2)
**Marriage**	Married	156 (31.2)
	Single	289 (57.8)
	Divorced	25 (5.0)
	Widow	14 (2.8)
	Lived Alone	6 (1.2)
	Missing data	10 (2.0)
**Education**	Bachelor	426 (85.2)
	Master	63 (12.6)
	Ph.D.	2 (0.4)
	Missing data	9 (1.8)
**Work in mental** **health ward**	1_5 years	205 (41)
	5–15 years	250 (50)
	16–30 years	28 (5.6)
	> 30 years	5 (1)
	Missing data	12 (2.4)

### POJ, MS, MC, MD, and burnout

The results indicate that the perceived level of POJ falls within the middle range overall (M = 2.25 ± 1.11, range: 1–5), including interactional, procedural, and distributive justice components. MS is also found to be in the middle range (M = 59.81 ± 13.77, range: 0-100). Similarly, MC is assessed as moderate overall (M = 53.16 ± 12.69, range: 15–105).

Furthermore, the findings reveal that participants experience moderate levels of MD (M = 53.16 ± 9.12, range: 0–90), with a stronger association with acquiescence to patients’ rights violations.

Table 3 presents the mean scores for the dimensions of burnout. Participants exhibit moderate burnout concerning client-related burnout, the nature work-related burnout, and work aversion-related burnout. However, personal burnout-related burnout scores are relatively low (M = 175.44 ± 56.63, range: 0-700), while other dimensions of burnout are at a moderate level.

**Table 3:** The distribution of MD, MC, MS, POJ and Burnout in the participants

Variables	Mean	SD
**POJ**	2.25	1.11
Interactional justice	2.68	0.70
Procedural justice	2.69	0.73
Distributive justice	2.70	0.78
**MS**	59.81	13.77
Moral behavior	21.02	5.33
Professional responsibility	19.13	4.53
Respect for the patient	19.70	5.22
**MC**	53.16	12.69
Moral agency	10.34	2.75
Multiple values	10.78	2.27
Endurance of threats	10.91	2.48
Going beyond compliance	10.60	2.51
Moral goals	10.45	2.38
**MD**	53.16	9.12
Acquiescence to patients’ rights violations	18.58	10.85
Unethical conduct by caregivers	14.70	8.43
Low staffing	11.90	6.83
**Burnout**		
Client related burnout	278.71	80.20
Work aversion related burnout	142.77	45.82
Nature work related burnout	146.79	48.29
Personal burnout	175.44	56.63

Abbreviations: SD, standard deviation; MD, Moral distress; MC, Moral courage; MS, Moral sensitivity; POJ, perceived organizational justice

### Interrelationships of variables

The relationship between variables is listed in Table 4; clearly there is a relationship between all variables except MS with personal burnout (r=-0.7, p = 0.12) and work aversion-related burnout (r = 0.03, p = 0.50); MC with personal burnout (r = 0.08, p = 0.07), and MD with nature work-related burnout (r = 0.07, p = 0.11). The strongest associations are between MD and POJ (r = 0.58) and MD and personal burnout (r = 0.56) among mental health nurses. Findings show the reverse relationship between POJ and all dimensions of burnout (P < 0.00).

**Table 4:** Pearson’s correlation of POJ, MS, MC, MD and burnout

Scale		POJ		MS		MC		MD		Burnout							
										Client related burnout		Work aversion-related burnout		Nature work-related burnout		Personal burnout	
		Pearson Correlation Coefficient	P- value	Pearson Correlation Coefficient	P- value	Pearson Correlation Coefficient	P- value	Pearson Correlation Coefficient	P- value	Pearson Correlation Coefficient	P- value	Pearson Correlation Coefficient	P- value	Pearson Correlation Coefficient	P- value	Pearson Correlation Coefficient	P- value
POJ		1		0.12	0.00^*^	0.16	0.00^*^	-0.58	0.00^*^	-0.41	0.00^*^	-0.30	0.00^*^	-0.39	0.00^*^	-0.45	0.00^*^
MS		0.12	0.00^*^	1		0.13	0.00^*^	0.18	0.00^*^	-0.27	0.00^*^	0.03	0.05	-0.29	0.00^*^	-0.07	0.12
MC		0.16	0.00^*^	0.13	0.00^*^	1		0.11	0.00^*^	0.09	0.00^*^	0.15	0.00^*^	0.13	0.00^*^	0.08	0.07
MD		-0.58	0.00^*^	0.18	0.00^*^	0.11	0.00^*^	1		0.23	0.00^*^	0.37	0.00^*^	0.07	0.11	0.56	0.00^*^
Burnout	Client related burnout	-0.41	0.00^*^	-0.27	0.00^*^	0.09	0.03^*^	0.23	0.00^*^	1		0.31	0.00^*^	0.55	0.00^*^	0.40	0.00^*^
	Work aversion- related burnout	-0.30	0.00^*^	0.03	0.50	0.15	0.00^*^	0.37	0.00^*^	0.31	0.00^*^	1		0.17	0.00^*^	0.49	0.00^*^
	Nature work-related burnout	-0.39	0.00^*^	-0.29	0.00^*^	0.13	0.00^*^	0.07	0.11	0.55	0.00^*^	0.17	0.00^*^	1		0.24	0.00^*^
	Personal burnout	-0.45	0.00^*^	-0.7	0.12	0.08	0.07	0.56	0.00^*^	0.40	0.00^*^	0.49	0.00^*^	0.24	0.00^*^	1	

^*^p < 0.05 (2-tailed)

### The path model

The hypothesized path model in Fig. 2 was tested, and the results showed inadequate model fitness (χ^2^=678.23, df = 127, p < 0.001, CFI = 0.91, RMSEA = 0.09). Acceptable values are as follows: > 0.95 for CFI, GFI, NFI; < 0.08 for RSMEA; < 0.06 for SRMR. The Table 5 indicated the CFI was 0.91, GFI was 0.87, NFI was 0.90; RSMEA was 0.093; and SRMR was 0.086. These fit indices imply an inadequate model fit (Table 5).

**Table 5:** The model fitness indices of moral distress in the participants based on Coley’s model

Indices^a^	χ^2^	df	χ^2^/df	P value	CFI	NFI	GFI	RMSEA	SRMR
Model	678.23	127	5.34	< 0.001	0.91	0.90	0.87	0.093	0.086

^a^ Acceptable values are as follows: > 0.95 for CFI, GFI, NFI; < 0.08 for RSMEA; < 0.06 for SRMR. The Table 5 indicated the CFI was 0.91, GFI was 0.87, NFI was 0.90; RSMEA was 0.093; and SRMR was 0.086, So, the results showed inadequate Coley’s model fitness

**Fig. 3:**
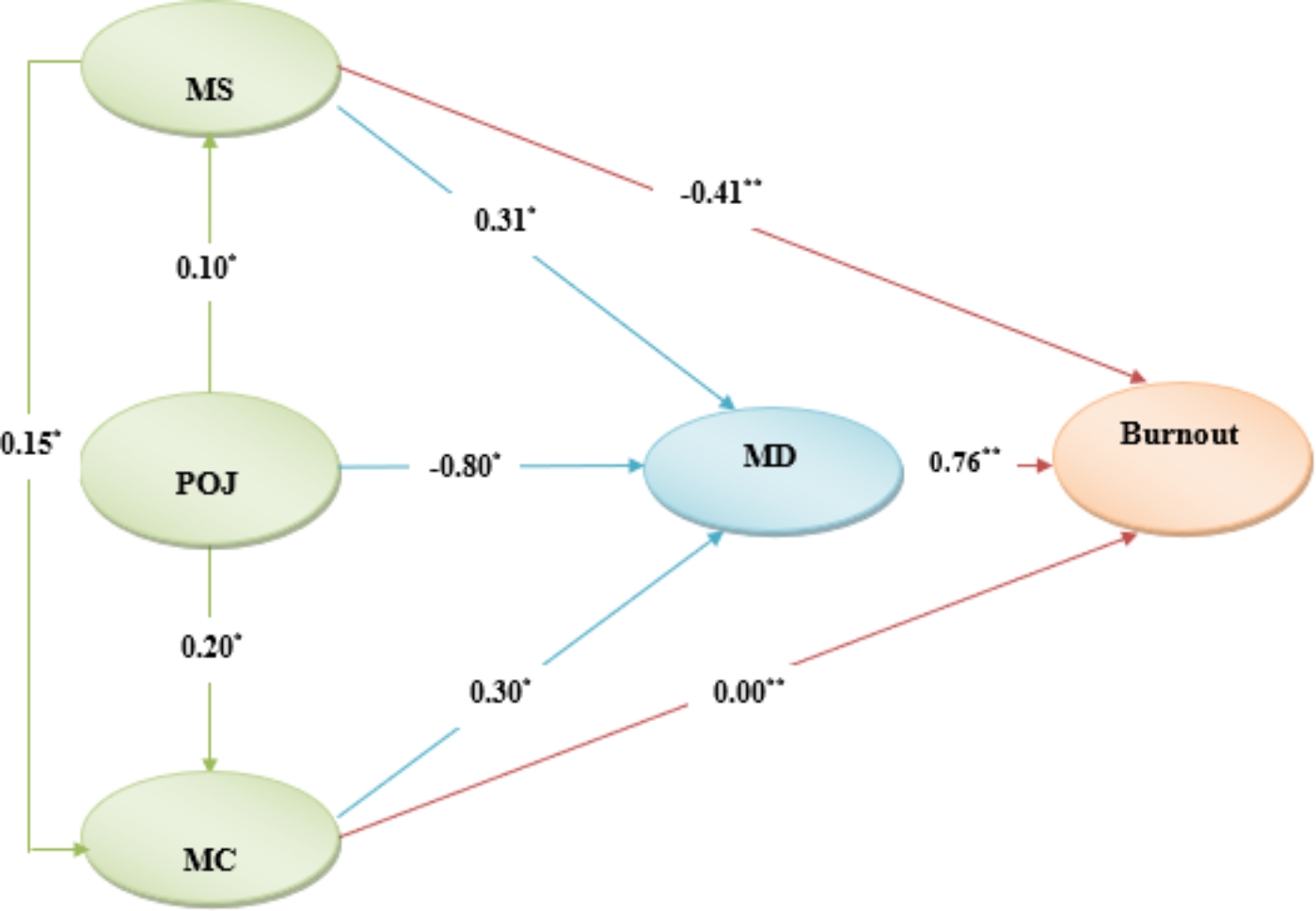
The examined path model (n = 500). POJ; Perceived Organizational Justice, MS; Moral sensitivity, MC; Moral Courage, MD; Moral Distress. *p=0.001. **p<0.05

As summarized in Fig. 3, the results show that the MD showed a strong direct and partial mediating effect on burnout in mental health nurses, implying that when MD was included, the relationship between the POJ and burnout (r = 0.82, β = 0.82) fell to a lower value. POJ had a strong and inverse standardized direct association with MD (β=-0.80, p = 0.001), and this unstandardized coefficient was − 0.58; revealing one-point increase in MD was associated with 0.58 decrease in POJ score. MD had a strong and positive direct association with burnout (β = 0.70, p = 0.001), and its unstandardized coefficient with personal burnout was 0.56; revealing one-point increase in MD was associated with 0.56 increase in personal burnout score.

## Discussion

This study aimed to provide a snapshot of the current state and examine the interrelationships of POJ, MS, MC, MD, and burnout based on Corley’s model of MD in mental health nurses.

### POJ, MS, MC, MD, and burnout scores

The present study’s variables are assessed at a moderate level, highlighting the importance of nurse leaders directing increased attention towards POJ, MS, MC, MD, and burnout. Consistent with our study, Haghighinezhad et al. [9] reported moderate levels of POJ in their findings. Conversely, studies conducted in Ethiopia [39], and in Japan [40] revealed low levels of POJ, differing from our current study.The staffing level, autonomy of nurses, and other conditions are different among countries, So the adverse results are taken for granted. POJ in mental health nursing refers to how these professionals assess fairness and equity in their workplace, especially concerning decision-making, resource allocation, and interpersonal interactions within the organization.

Our research indicates that MS among mental health nurses is at a moderate level. This is consistent with a study by Mahdaviseresht et al. [24], which also reported moderate MS. However, in contrast, a study conducted by Mohammadi et al. where the participants were critical care nurses [33], and Khodaveisi et al., whose study was conducted to nurses dealing with COVID-19 [41], found that nurses experienced high levels of MS. Variations in MS levels among mental health nurses, as revealed in present research, may be primarily attributed to the distinctive nature of their profession. Unlike critical care or general nursing, mental health nursing confronts intricate ethical dilemmas concerning patients’ mental well-being and rights. These issues are less prevalent in other nursing specialties. Differences in workplace cultures and patient demographics also contribute to MS level disparities in various studies. Furthermore, the lack of prior research on MS in mental health departments underscores a literature gap, indicating an underexplored aspect of nursing ethics.

Our findings are same as one study in China [42], indicate that MC is at a moderate level among mental health nurses, which contrasts with the higher levels reported by Pajakoski et al. [11], Mahdaviseresht et al. [24], Khodaveisi et al. [41], Mohammadi et al. [43], Ebadi et al. [44], and Pakizekho et al. [45]. MC helps nurses to overcome many obstacles like fear and as a result can advocate patients in a good manner. One rational reason for the difference in MC levels among mental health nurses in our research compared to those in other departments, as indicated in our research findings, could be the unique nature of challenges and ethical dilemmas faced by mental health nurses. Mental health nursing often involves complex ethical situations related to patient autonomy, involuntary treatment, confidentiality, and managing potential harm to oneself or others. These nurses may confront moral dilemmas that are distinct from those encountered in other healthcare settings. As a result, their perception and expression of MC might differ, leading to the observed moderate levels in contrast to studies conducted in different departments where the ethical considerations and challenges could be distinct. The variations in ethical demands across healthcare specialties can reasonably account for the differences in reported levels of MC.

The current research findings indicated that nurses’ scores for MD were generally average. Some studies [3, 9, 46], reported moderate MD levels, while others [22, 47, 48] reported MD levels higher than moderate. These inconsistencies may be attributed to various factors, such as organizational, cultural, educational, geographical, and individual differences, as well as differing beliefs among participants. Additionally, variations in healthcare standards, knowledge, staff engagement, and ethical qualities may contribute to these differences. The study’s population and measurement instruments may also play a role in the observed disparities.

The present results revealed that all dimensions of burnout (except personal burnout dimension) were at a moderate level, which aligns with the findings from several studies conducted in Egypt [49–52]. This finding is consistent with the results of a Meta-analysis study, which reported that burnout among mental health nurses, is at low to moderate level [20]. Some of other researchers such as Shoorideh et al. [3], López-López et al. [20], Scanlan and Still [53], and Tsai et al. [54] reported burnout is moderate level. Georges et al. reported low levels of burnout among American nurses [55], while scholars have presented varying findings regarding the extent of burnout in different units in Iran. In contrast to the present study, Salimi et al. [56] and Tavakoli et al. [57] found a high level of burnout. Similarly, in countries such as China [58], and Saudi Arabia [59], burnout among nurses was found to be high. Significant differences were observed in all aspects of burnout among different wards, consistent with numerous similar studies conducted on nurses working in various wards. However, comparing results is challenging due to the use of different assessment tools and unique study samples. Burnout appeared to be a prevalent issue among participating nurses, emphasizing the need to identify and address sources of burnout in hospitals. Furthermore, teaching nurses coping strategies to manage burnout is deemed an essential step in mitigating this problem.

Professionals working in mental health settings often confront a heightened risk of burnout owing to the demanding nature of their roles. This includes managing a heavy caseload, limited avenues for career progression, and relatively modest financial compensation. Furthermore, these dedicated individuals regularly engage with patients who may display aggressive, violent, or suicidal tendencies, intensifying their exposure to job-related stress and compassion fatigue [60]. Burnout among mental health nurses can have a profound impact, not only on the well-being of the individual nurses but also on the quality of care provided to those under their supervision. Consequently, there is a pressing need for additional research in this field and the development of effective strategies to mitigate burnout among nurses.

### Relationship between POJ with MS

The relationship between POJ and MS in nurses demonstrated a significant connection. This aligns with Rodwell et al.‘s findings [14], which indicated that higher levels of POJ may contribute to increased MS among nurses, consistent with our own research. This relationship likely exists because nurses who perceive fairness and justice in their workplace tend to develop heightened moral sensitivity, enabling them to be more attuned to ethical issues and dilemmas in patient care.

### Relationship between POJ with MD

Same as our findings, Haghighinezhad et al. found a significant negative correlation between the POJ and the MD, and between “procedural and interactional justice and errors” with “not respecting the ethics principles” [9]. Most likely, this negative relationship arises because when nurses perceive fairness and equity in their workplace, they are less likely to experience moral distress, which often results from situations involving perceived unfairness, ethical dilemmas, or conflicts with organizational policies or practices.

### Relationship between MS with MC

The relationship between MS and MC in nurses working in mental health wards was significant, while the relationship of MC with respect for patient, professional responsibility (dimensions of MS); and the relationship of MS with moral goals and exceeding compliance (dimensions of MC) were not significant. Mahdaviseresht et al. [24], Hemati et al. [61], Mohammadi et al. [43] in Iran, and Escolar-Chua [62] in Philippine reported that there was a significant relationship between MC and MS. Besides in a recent study, researchers found a significant and positive link between MC, MS, and the provision of safe nursing care [41]. Nurses with higher MS are more aware of wrongdoings and insufficiency in patient care. It is likely that they feel compelled to act as advocates for patients, which leads to higher MC.

### Relationship between MS with MD

The results indicate a significant relationship between MS and its dimensions with MD dimensions. Ohnishi et al. showed that nurses with high MS suffer from MD [35]. Milliken argued that individuals who do not have sufficient executive powers to perform ethically suffer from MD, regardless of their MS [63]. In addition, Lützén and Kvist (2012) showed that individuals who are not allowed to make their own moral decision, feel a higher level of MD regardless of their MS level [64]. As a result, nurses with elevated MS but lacking adequate executive authority face an increased risk of MD when confronted with morally stressful situations [65].

### Relationship between MC with MD

This research findings revealed a significant relationship between MC and MD. Nurses with a high MC are likely to make courageous decisions in the face of moral challenges, but without enough powers they will fail to achieve their goals, which leads to MD. Mohammadi et al. [66], and Safarpour et al. [28] found this positive relationship. Inconsistent with our study, Karampourian et al. [46] also identified the inverse relationship between MC and MD. The controversy may stem from differences in research methodologies, such as measurement tools or data collection techniques, between the current study and previous research. These methodological variations can contribute to conflicting results and challenge the comparability of findings. Non-mental health nursing and mental health nursing may involve distinct contexts, patient populations, and ethical dilemmas. These contextual differences could lead to variations in how MC and MD manifest in these settings, further fueling the controversy.

### Relationship between MS with Burnout

Our findings on the relationship between MS and burnout showed that MS only had a significant relationship with nature work-related burnout and client-related burnout. In addition, there was a significant relationship between client-related burnout and respect for the patient, professional responsibility, and ethical behavior (dimension of MS). There was a significant relationship between personal burnout and professional responsibility and there was a significant relationship between nature work-related burnout and respect for the patient. There was no relationship between work aversion-related burnout and the MS dimensions. In line with our findings, Palazoğlu et al. (2019) found an inverse and significant relationship between burnout and MS among nurses in Turkey [67]. The nature of work and patient interactions in mental health nursing can differ significantly from other nursing specialties. This variation in context may result in different patterns of association between MS and burnout. Mental health nurses may face unique challenges and stressors that influence the relationship between these variables. Also, Cultural norms, healthcare policies, and organizational practices specific to mental health nursing may impact the observed relationships.

### Relationship between MC with Burnout

There was no significant relationship between MC and personal burnout, whereas MC had significant relationship with other dimensions of burnout. On the other hand, the relationship between nature work-related burnout and dimensions of MC was inverse and not significant, while the relationship between nature work-related burnout and MC was direct and not significant. In light of this study, the findings from Zakeriafshar et al. in 2023 indicate that there was no statistically significant relationship between burnout and MC [68]. Also, in one study by Alshammari et al., revealed that MC played a significant role in mediating the indirect impacts of burnout and professional competence on compassion fatigue [69]. To explain the findings, the nature of mental health wards inevitably can cause stress and tension among nurses, which in the long term can lead to burnout and professional dissatisfaction, absenteeism from work or higher turnover. Therefore, MC helps nurses to overcome their fear and limitation and make the right decision in any situation.

### Relationship between POJ with burnout

The findings of current study show a reverse relationship between POJ and Burnout. In line of current study, the results of the study conducted by Kim et al. indicate that interpersonal and procedural justice play a pivotal role in alleviating burnout [70]. Based on the study conducted by Elçi et al., it was observed that POJ has a detrimental impact on burnout [71]. The findings provide compelling evidence supporting the inverse correlation between POJ and the occurrence of burnout [70, 71]. Most likely, the negative relationship between perceived organizational justice and burnout arises because when nurses perceive fairness and equity in their workplace, they are less likely to experience burnout.

### Relationship between MD with burnout

The results showed that MD was significantly related with personal burnout, work aversion-related burnout, and client-related burnout. However, there was no significant relationship with nature work-related burnout. Delfrate et al. (2018) studied nurses in a mental health ward and showed a significant and direct relationship between burnout and MD [72]. The results of Escolar-Chua showed that MD and all its dimensions had a positive and significant relationship with personal burnout, nature work-related burnout, and client-related burnout [62]. Shoorideh et al. (2015) showed a positive correlation between MD errors and nature work-related burnout and a significant relationship between MD and client-related burnout [3]. Shafie et al. (2015) showed a positive and significant relationship between intensity and frequency of MD and burnout [73]. An integrative review of the literature reveals the consequences of MD on the nursing workforce particularly regarding nurses’ burnout [74]. These studies are consistent with the present work except in nature work-related burnout dimension.

Because the nature work-related burnout in mental health nursing is different from other departments, such as intensive care units, it can be concluded that there is no significant relationship between MD and nature work-related burnout. The explanation for this finding is that nurses tend to perceive higher job stability as they gain more experience in their work environment, which can lead to a correct perception of the quality of their work life. The extent of MD and burnout depends on the work environment and personal characteristics, so emotionally sensitive individuals are more susceptible to the patient condition and experience higher levels of MD. In addition, conflict and insecure relationship with managers may increase MD in nurses.

### Model fitness

As the results showed, MS, MC, and MD (mediated by POJ) had a direct influence on burnout in nurses. Therefore, nurses need to pay more attention to these factors to improve the quality of care along with the goals and mission of hospitals. The participants were nurses working in hospitals; therefore, the results can be used to improve nurses’ work environment, increase job satisfaction, and decrease burnout.

This model comprises five factors, and even more if we include subscales, making it quite intricate. Furthermore, MC operates in two distinct ways: it can empower nurses to overcome MD, but it may also push them to act recklessly, potentially leading to failure and subsequent Moral Distress.

## Conclusions

This study delved into the intricate web of relationships among variables derived from Corley’s model within the context of mental health nursing. The overarching aim was to explore how POJ, MS, MC, MD, and burnout interconnect in the professional lives of nurses.

Building upon Corley’s model, which posits that burnout and MD are outcomes influenced by a complex interplay of organizational and individual factors, our investigation extended the scope to encompass MC and MS as key individual traits. These traits were explored alongside POJ, regarded as an antecedent, and the consequences of MD, namely burnout.

Our study identified an inverse relationship between POJ and MD. It suggests that when nurses perceive greater organizational justice, they may experience lower levels of MD. In contrast, MS and MC displayed a direct relationship with MD, implying that higher levels of these traits may correspond with increased MD.

However, it is essential to acknowledge that our study also indicated inadequate model fitness. This highlights the complexity and multifaceted nature of the relationships under investigation. While our findings provide valuable insights into the interconnectedness of these variables, they also underscore the need for further exploration and refinement of Corley’s model with additional variables and in various healthcare contexts.

In summary, our study contributes to the growing body of knowledge surrounding the experiences of mental health nurses and their interactions with organizational and individual factors. The relationships among POJ, MS, MC, MD, and burnout are nuanced and multifaceted. As we continue to advance our understanding of these dynamics, we pave the way for interventions and strategies aimed at improving the well-being of nurses and the quality of care they provide in mental health settings. Further research and continued efforts to refine and expand research model will be essential in addressing these complex challenges in healthcare.

### Limitations

Our study exhibits several limitations. Firstly, our research methodology did not permit an examination of the random effects of the variables, necessitating the need for future experimental studies to address this limitation. Additionally, due to the intricate nature of numerous variables, certain elements of Corley’s model of Moral Distress were isolated in our study, limiting a comprehensive analysis.

Furthermore, a lack of prior research on the measurement and relationships of these variables among mental health nurses hindered our ability to make comparisons with previous studies. Lastly, it is important to acknowledge that the generalizability of our study’s findings is restricted by the specific context, organizational policies, and the prevailing atmosphere concerning moral issues among nurses.

### Electronic supplementary material

Below is the link to the electronic supplementary material.

Supplementary Material 1

## Data Availability

All data generated or analyzed during this study are included in this article and its supplementary file.

## Abbreviations

AIC: Average Inter-item Correlations
df: Degrees of Freedom
ICC: Intra Class Correlation
MD: Moral Distress
MC: Moral Courage
MS: Moral Sensitivity
POJ: Perceived Organizational Justice
SEM: Structural Equation Model
CFI: Comparative Fit Index
GFI: Goodness of Fit Index
NFI: Normed Fit Index
RMSEA: Root Mean Square Error of Approximation
SRMR: Standardized Root Mean Squared Residual
